# Smartphone-Assisted Medical Care for Vestibular Dysfunction as a Telehealth Strategy for Digital Therapy Beyond COVID-19: Scoping Review

**DOI:** 10.2196/48638

**Published:** 2023-09-11

**Authors:** Masao Noda, Tatsuaki Kuroda, Akihiro Nomura, Makoto Ito, Tomokazu Yoshizaki, Hiroaki Fushiki

**Affiliations:** 1 Department of Otolaryngology Jichi Medical University Shimotsuke Japan; 2 Mejiro University Ear Institute Clinic Saitama Japan; 3 Kuroda Ear, Nose and Throat Clinic Kumamoto Japan; 4 College of Transdisciplinary Sciences for Innovation Kanazawa University Kanazawa Japan; 5 Department of Otolaryngology Kanazawa University Kanazawa Japan

**Keywords:** dizziness, vertigo, telemedicine, smartphone, digital therapy, telehealth, COVID-19, information technology, scoping review, health device, remote diagnosis, medical care

## Abstract

**Background:**

Dizziness and vertigo can be caused by various factors, such as peripheral vestibular and central disorders. Although consultations with specialists are advisable when necessary, patients with severe vertigo symptoms may have limited mobility, which may interfere with hospital visits. The spread of COVID-19 has further limited the number of hospital visits for patients with dizziness; therefore, a method of medical care that enables more accurate treatment under time and geographical constraints is needed. Telemedicine has become widespread, owing to the popularity of smartphone and tablet devices in recent years, and the use of devices and systems has made it possible to provide efficient medical care. However, no previous scoping review has mapped existing studies on telemedicine for vertigo and dizziness, and no recommendations have been made regarding which devices and systems should be used for specific diseases.

**Objective:**

The aim of this review was to map and assess previous studies on the use of information communications technology, smartphones, and apps for treating patients with vertigo and discuss the added value of introducing telemedicine to improve the quality of medical care and create an environment that builds security and trust among patients.

**Methods:**

A scoping review was conducted with the methodological framework of Arksey and O’Malley and in accordance with the of the PRISMA-ScR (Preferred Reporting Items for Systematic Reviews and Meta-Analysis extension for Scoping Reviews) guidelines. The PubMed, MEDLINE, and Cochrane Library databases were searched to retrieve previous reports on smartphone-assisted telemedicine treatment for vertigo published between January 2000 and May 2023. Two authors independently assessed eligibility and extracted data.

**Results:**

This review included 20 papers that reported devices or systems for telemedicine for vestibular dysfunction. Among studies that reported the use of a device or app, 2 were related to anamnesis and subjective symptoms, 12 were related to objective examination, 7 were related to remote diagnosis, and 7 were related to treatment and rehabilitation.

**Conclusions:**

With the advancement of technology, the use of telemedicine in patients with dizziness may be feasible. In the future, it will be necessary to consider how telemedicine can be used in dizziness treatment and develop an effective treatment system combining in-person medical care and the effective use of devices for the management of severe vertigo and related diseases. The smooth introduction of telemedicine in vertigo treatment is expected to improve the quality of treatment, increase opportunities for patients to receive medical care, and reduce time and travel costs, leading to a sense of security and trust among patients.

## Introduction

Dizziness and vertigo are common symptoms experienced by people of all ages, with lifetime prevalence estimates of 17%-30% for dizziness and 3%-10% for vertigo [1]. Dizziness and vertigo can be caused by various factors, including peripheral vestibular disorders, central disorders, circulatory dysfunction, headache-related vertigo, and psychogenic vertigo, which transcend the framework of medical departments. Among various cases of dizziness, differentiating between peripheral vertigo and stroke is clinically important for the early detection of a life-threatening condition. However, patients with severe vertigo symptoms may have limited mobility, which may interfere with hospital visits. In addition, the diagnosis may be delayed or prevented as the dizziness symptoms and significant clinical findings have subsided or are no longer available at the time of the hospital visit. Thus, a system to inform patients of their symptoms and findings outside the premises of the hospital is necessary to improve the accuracy of medical care and make an early diagnosis.

In recent years, telemedicine has become widespread owing to the popularity of smartphone and tablet devices. Moreover, the development of data communication technologies and the use of devices and systems for medical care has made it possible to provide efficient medical care [2-5]. Establishing a way to acquire observations remotely in cases of medical emergencies, such as stroke or arrhythmia during the acute phase, will lead to effective medical treatment methods in the future. The COVID-19 pandemic has significantly impacted the diagnosis and treatment of dizziness or vertigo. The spread of COVID-19 has limited the number of hospital visits for patients with dizziness, and many physicians are searching for newer methods to treat dizziness during the pandemic. Interestingly, Barreto et al [5] have set a new direction for vestibular evaluation using smartphones for the diagnosis of vestibular hypofunction and mentioned technical conditions and limitations for patient privacy. However, no guidelines have been established for treatment strategies for vertigo and dizziness, and no recommendations have been made regarding which devices and systems should be used for which diseases. Furthermore, a strategy for treating vertigo that works in a combined approach must be developed, rather than just an isolated examination or consultation method using smartphones.

With the rapid spread of telemedicine, there is a need to conduct an updated and broader literature review to develop an overview of the body of knowledge within this field. To our knowledge, no scoping review has mapped existing studies on telemedicine for vertigo and dizziness, and no recommendations have been made regarding specific devices and systems that should be used for various diseases.

Herein, we aim to map and assess previous studies on the use of information communication technology (ICT), smartphones, and apps for treating vertigo patients by reviewing previous reports on telemedicine for treating patients with vertigo using smartphones or apps.

## Methods

### Overall Study Design

A scoping review was performed using PRISMA-ScR (Preferred Reporting Items for Systematic Reviews and Meta-Analyses extension for scoping review) guidelines [6].

The review subsequently proceeded with the stages using the framework given by Arksey and O’Malley [7]: identifying the research question and relevant studies; selecting studies; charting the data; and collating, summarizing, and reporting the results. The protocol for this scoping review has not been registered or published.

### Search Strategy

We conducted a search of medical literature databases (MEDLINE via PubMed, Cochrane Library) to identify relevant reports for this scoping review on smartphone-assisted treatment for vertigo telemedicine. The search was conducted between January 1, 2000, and May 30, 2023.

The search strategy was developed by 3 authors (TK, MN, and HF) and applied to each database. A set of controlled variables (“dizziness” or “vertigo” and “telemedicine” or “teletreatment” or “Internet” or “diagnosis” and “smartphone” or “cellphone” or “application” or “Digital Therapeutics”) were used to identify relevant studies. In addition, a manual search was performed to screen the reference lists of the included papers.

All identified studies were reviewed to determine whether they answered the following questions: “How can a smartphone be used in dizziness telemedicine?” and “What types of devices and applications have been reported to be related to dizziness, especially for monitoring, testing, teleconsultation, and therapy?” We applied specific inclusion and exclusion criteria based on study type, period, type of materials, target population, and type of medical act (Textbox 1). The authors independently screened titles, abstracts, and full-text papers for inclusion in the study.

**Inclusion and exclusion criteria.**

**Inclusion criteria**

- Qualitative, quantitative, and mixed methods studies published in peer-reviewed journals
- From January 1, 2000, to May 30, 2023
- All languages
- Use of web apps, smartphones, and devices for participants
- Use of materials related to dizziness and vertigo for patients
- Use of materials for participants at the stages of anamnesis, symptoms, objectives, diagnosis, and treatments

**Exclusion criteria**

- Conference abstracts, doctoral theses, and reviews
- Before January 1, 2000
- No use of devices or smartphones or the internet
- Use of materials for diseases not related to dizziness
- Introduction of devices and functional test of devices

### Charting Data

Two investigators (MN and HF) independently extracted study-specific data using a standardized collection form with the following information: authors, year of publication, title, aim, telehealth device or app, design and methods, timing of material use, and summary.

### Summarizing and Reporting Results

We used an inductive approach to address the research question, focusing on themes identified from the included papers. The results were summarized accordingly. The types of medical acts were divided into 4 thematic groups: anamnesis and subjective symptoms, objective tests, diagnosis, and treatment.

## Results

### Overview

**Figure 1:**
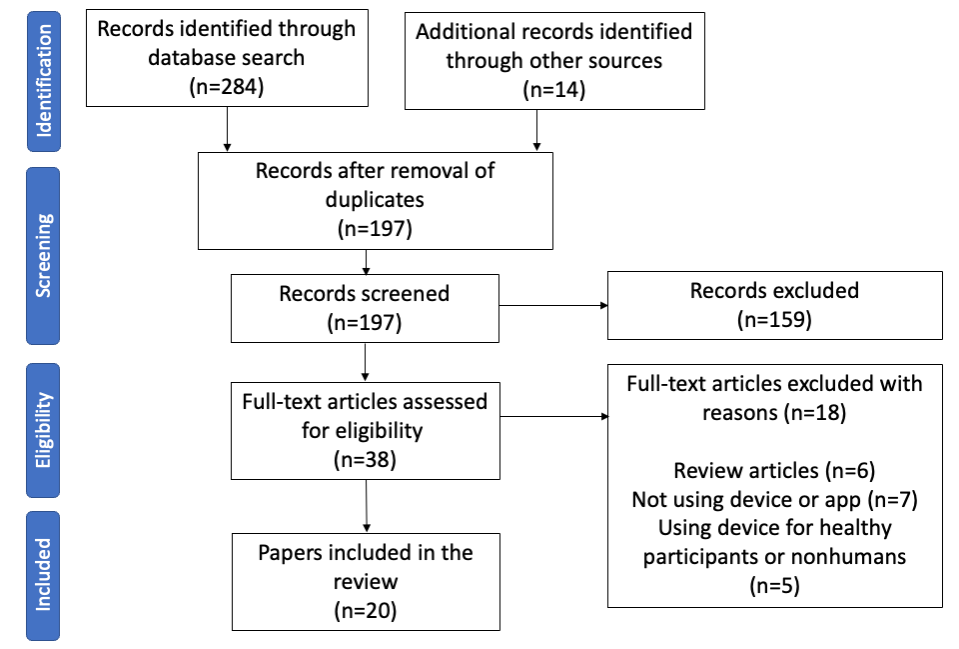
PRISMA-ScR (Preferred Reporting Items for Systematic Reviews and Meta-Analyses) flowchart of the systematic search strategy.

The database and manual searches yielded 356 publications. After the removal of duplicates, the titles and abstracts of 289 publications were screened. Based on the inclusion and exclusion criteria, the full texts of 38 publications were read, 18 publications were excluded, and 20 publications were included in this review (Figure 1, Multimedia Appendix 1 [3,8-26]).

Among the 20 studies that reported the use of a device or app, 2 were related to anamnesis and subjective symptoms [3,8], 12 were related to objective examination [8-19], 7 were related to remote diagnosis [9,11,15,16,18,20], and 7 were related to treatment and rehabilitation [20-26] (Table 1).

**Table 1:** Articles included in thematic groupings.

Theme	Study	Articles, n
Used for anamnesis and subjective symptoms	Jaensson et al [3] Martin et al [8]	2
Used for objective tests	Martin et al [8] Kiroglu et al [9] Jacob et al [10] Gold et al [11] Phillips et al [12] Parker et al [13] Ippisch et al [14] van Bonn et al [15] Wagle et al [16] Riera-Tur et al [17] Shah et al [18] Wengier et al [19]	12
Used for diagnosis	Kiroglu et al [9] Gold et al [11] Phillips et al [12] van Bonn et al [15] Wagle et al [16] Shah et al [18] Bamiou et al [20]	7
Used for treatment	Bamiou et al [20] Tabanfar et al [21] van Vugt et al [22] Kanyılmaz et al [23] Guerrero et al [24] Soto-Varela et al [25] Lubetzky et al [26]	7

All papers included adult patients. Seven papers were randomized controlled trials [3,9,20-23,25]. Ten papers used a mobile phone, among which 5 included an app for the subjective visual vertical (SVV) test [10,17,19], head impulse test (HIT) [13], and hearing test [8]. Three papers used virtual reality (VR) for treatment with the Epley maneuver [21] and vestibular rehabilitation [23,26]. Eight papers included apps or devices with more than one category of medical act performed; all papers were about sequential acts [8,9,11,12,15,16,18,20].

To answer the research question, “What types of devices and applications have been reported to be related to dizziness, especially for monitoring, testing, teleconsultation, and therapy?” the results of this review are presented in 4 thematic groupings: anamnesis and subjective symptoms, objective test, diagnosis, and treatment.

### Anamnesis and Subjective Symptoms

Two papers reported the use of mobile phone apps for assessing anamnesis and subjective symptoms. Both systems involved the input of daily symptoms by patients, which were then reviewed by a medical provider who monitored the postoperative symptoms and severity of symptoms related to dizziness [3,8]. Postoperative patient follow-up led to reassurance and positive feedback, and postoperative dizziness symptoms improved significantly [3]. Subjective symptoms and self-hearing tests were recorded in combination with a hearing test [8].

The severity of self-reported tinnitus increased significantly with the increase in the severity of self-reported hearing loss in the affected (*P*=.01) and unaffected ears (*P*<.001) and was useful in determining the severity of Ménière disease.

### Objective Test

Twelve papers reported the use of mobile phone apps for objective tests. Seven of the reports used examination data to diagnose various diseases, including acute and chronic vertigo, in the emergency department [9,11,18]. In contrast, only 1 study combined these with subjective symptoms (Martin et al [8]) and used Dizzy Quest and an iPad-based hearing test.

Various types of tests are conducted in vertigo treatment, including balance function, nystagmus, and visual stimulation tests, such as SVV. The types of tests in the report included HIT [13], SVV [10,17,19], nystagmus [9,11,12,15,16,18], gait analysis [14], and hearing tests [8].

For nystagmus, some researchers used the camera functionality of smart devices and directly shared their videos, while others used deep learning to classify the video data [16].

In a report comparing remote and existing examinations, Parker et al [13] reported a video HIT app (iPhone) that uses the iPhone’s Augmented Reality Kit system. Shah et al [18] reported that the remote screening of benign paroxysmal positional vertigo (BPPV) is possible with high specificity. The sensitivity for diagnosing BPPV via a smartphone recording of eye movements of the Dix Hallpike test was 92.86%, with a specificity of 100% and a negative predictive value of 97.87%.

### Diagnosis

Among the included papers, 7 reported the use of mobile phone apps for the diagnosis of dizziness. These apps were used after conducting objective tests, and they focused on identifying central vertigo, BPPV, and Ménière disease [9,11,18]. All of these studies used smartphone apps as diagnostic tools.

Another paper reported the EMBalance decision support system (DSS), a multilanguage platform, for diagnosing vestibular disorders in primary care settings [20]. The EMBalance DSS serves as a supportive tool by providing a structured and detailed diagnostic and management plan for various vestibular disorders. In terms of diagnostic accuracy, the study found that nonspecialist physicians using the EMBalance DSS had a higher percentage of correct diagnoses compared with those without the DSS (54% vs 41.5% correct diagnoses, respectively). As a standalone tool, the DSS demonstrated better sensitivity for first- and second-line diagnostic decisions compared with the nonspecialist group without the DSS (odds ratio 3). However, it is important to note that the specificity of the DSS was somewhat weakened.

### Treatment

Seven papers reported the use of mobile phone apps or the internet for the treatment of dizziness. Physical therapy, such as head position therapy and rehabilitation, was identified as an effective treatment for vertigo. Among these 6 papers, 2 used VR technology, the Epley maneuver [21], and vestibular rehabilitation [23,26]. Both studies reported long-term therapeutic effects over a 6-month period. In addition, van Vugt et al [22] reported more effective results when the mobile phone app was incorporated into regular rehabilitation. Guerrero et al [24] examined the effects of a virtual exercise program on balance in adults with Down syndrome and reported a significant improvement compared with the pretesting level.

## Discussion

### Principal Findings

This scoping review mapped and assessed previous studies on the use of ICT, smartphones, and apps for treating patients with vertigo. The results demonstrated that smartphones and apps were feasible for use in dizziness telemedicine and enabled high-quality dizziness care at home and other remote locations, resulting in a practice that benefits both physicians and patients. Dizziness is a symptom perceived by people of various ages. Approximately 30% of adults aged >65 years report experiencing dizziness that interferes with their daily life [27]. Patients with dizziness are at an increased risk of falling, which interferes with daily life and limits their participation in social activities [27,28]. In addition, the time available for in-person medical care is limited in the clinic, and there are many geographical constraints. To use telemedicine and teleconsultation for patients with dizziness, considering a variety of symptoms and tests that feature in dizziness treatment and an effective combination of individualized interviews, examination, and treatment methods could lead to the development of an effective treatment strategy.

In this review of articles published since 2000, most were published after 2015, indicating an increasing trend in the need for telemedicine over the years.

Many reports on telemedicine for vertigo were published around 1990. Wolf et al [29] reported “telemetric” electronystagmography, which involved conducting nystagmus testing outside of the clinic setting. Viirre et al [30,31] reported on efforts to use a head mount device for nystagmus evaluation and VR technologies in rehabilitation. As many mechanical tests, such as nystagmus, balance function, and hearing tests, are used in clinical practice for vertigo, the medical care approach is influenced by the evolution of telecommunications and devices. Furthermore, the demand for telemedicine has been increasing since the late 2010s due to the widespread use of smartphones, internet connectivity, and the global COVID-19 pandemic. With the advent of innovative methods, there is a need to discuss the effectiveness and challenges associated with devices in vertigo management and the optimal use of these tools.

Regarding the current theoretical and practical research gaps, the use of smartphones and apps allows for a variety of medical treatments equivalent to existing dizziness treatments. Studies have been conducted on several diseases, their use in acute and chronic phases, and their application to examination, as well as diagnosis, symptoms, and rehabilitation. However, there is a lack of comprehensive use of each app and device in combination and a lack of examples of use for diagnosis and interviewing. These limitations may be attributed to the following reasons: multiple systems are not yet well coordinated, which makes medical treatment time consuming; the IT literacy of users; the development of an environment in which the systems can be used; and the medical care system, including insurance coverage.

Regarding the use of devices in the papers reviewed, most of the cases used the device for objective examination rather than other medical acts. There were a few cases of device use for multiple medical acts; however, there were no reports of device use for multiple acts, such as anamnesis-to-diagnosis or examination-to-treatment.

Dizziness is caused by various pathological conditions in the inner ear, nervous system, and musculoskeletal system. Consequently, a combination of tests, including nystagmus evaluation, balance function assessment, and imaging techniques (such as computed tomography and magnetic resonance imaging), is required for thorough examination and diagnosis. Therefore, smartphones and other devices are being increasingly used as substitutes for conventional testing methods, facilitating the diagnostic process for patients with vertigo. By combining various devices and apps, it is feasible to build a comprehensive system for vertigo treatment, and the future holds great promise.

VestAid is an innovative tablet-based system for vestibulo-ocular reflex exercises [32]. VestAid validated eye-gaze accuracy as part of the study and detected eye movement abnormalities in the participants with directed energy exposure, concussion, and vestibular neuritis [33]. Although not included in the review as this paper is in the initial evaluation stage of the system, this system is capable of recognizing facial movements and providing rehabilitation for vertigo through games. VestAid can also perform objective and subjective data collection and is expected to be used for multiple medical acts in various apps in the clinical practice of vertigo.

In the management of dizziness, subjective symptoms and objective findings are used for diagnosis, and in some cases, the final diagnosis depends on the response after treatment. Thus, comprehensive dizziness care leads to improvement in the quality of care. For chronic dizziness, diagnosis may be confirmed by excluding other diseases, depending on the accompanying symptoms and their severity. Among individuals under the age of 20 years, migraine-associated dizziness, including vestibular migraine and benign paroxysmal vertigo of childhood or orthostatic dysregulation, are the most common types of vertigo; however, there are cases in which the two overlap, and the diagnosis is unclear. There have been several reports of anxiety and fear, anemia, menstruation, hypotension, insomnia, headache, stiff shoulders, and neck, with coexisting or overlapping menopausal symptoms in women with dizziness. It is essential to monitor dizziness and lightheadedness during daily activities in such cases.

For diagnosis using a smart device, diseases related to the diagnosis or exclusion were central vertigo identification, BPPV, and Ménière disease [9,11,18]. The dizziness experienced by the patient is mild in many cases of recurrent dizziness, such as Ménière disease, delayed endolymphatic hydrops, vestibular migraine, and BPPV, and there are no significant findings at the time of presentation, making a definitive diagnosis difficult. Therefore, eye movement findings during a vertigo attack are crucial for a definitive diagnosis. Kiroglu et al [9] reported the use of a cell phone camera for diagnosing Ménière disease. Compared with the conventional method of diagnosis, no significant differences were observed in the rate of diagnosis of Ménière disease when recording eye movement during a vertigo attack [9].

There have also been several instances of artificial intelligence implementation in nystagmus and vestibular rehabilitation using VR. The accuracy of video-oculography using a smartphone has shown steady improvement. Parker et al [34] and Friedrich et al [35] reported, respectively, that ARkit-based apps and ConVNG are developing methods ranging from pupil detection to nystagmus analysis, which are expected to further improve the accuracy of diagnosis in smartphone vertigo practice [34,35].

Examination findings in vertigo treatment are often judged by referring to waveforms and images, which are also quantified, making it a suitable field for machine learning and deep learning. In addition, medical devices that monitor eye movements, such as Frenzel glasses, are similar to VR goggles and could be introduced to both physicians and patients without any significant modifications. With technological advancements, the quality and accuracy of vertigo medical care are expected to improve further.

Concerning the target users of the system, most apps were used by patients, and the data were confirmed by the medical staff. The current style of medical care mainly involves interaction between physicians and patients. There is an effort to change this style, as enhancing collaboration between patients and physicians as well as between physicians is expected to further expand the range of medical care that can be provided. Physician-to-patient telemedicine has many advantages, such as the reduction of time and travel costs, reduction in the waiting period for medical care, increased opportunities to see patients, avoidance of risk of spread of infection during pandemics, and complementary medical care in the event of a patient becoming a high-risk contact.

The EMBalance DSS developed by Bamiou et al [20] describes a system in which primary care physicians consult with specialists. EMBalance is effective both as a supporting tool and a standalone tool, creating an opportunity for appropriate medical care in the absence of specialists. Balance disorders can occur due to various causes, including peripheral vertigo caused by the inner ear and vestibular nerves, problems in the cerebellum and brainstem, circulatory dysregulation (ie, arrhythmia), and dizziness related to migraines, anxiety, and depression. Although circumstances may vary by region, this type of consultation between general physicians and specialists may be necessary. Nevertheless, specialists in dizziness are unevenly distributed, and there is a possibility of disparities in the medical care they can provide. Telemedicine enables collaboration between physicians and patients, as well as among physical therapists, pharmacists, and patients’ families. Regarding the execution of rehabilitation and sharing of medical conditions, the geographical limitations of conventional in-person consultations will be eliminated.

There are challenges and limitations in the dissemination of dizziness telemedicine, such as privacy, communication environment, information leakage, and ICT literacy. Although it is technically possible to provide medical care equivalent to in-person consultation, it is necessary to clarify the safety and legal issues related to apps and devices. Another challenge is differentiating between acute and emergency dizziness.

Serious conditions must be ruled out in the emergency department as telemedicine is not always able to respond to emergencies for which contact is required. It is important to differentiate between BPPV, Ménière disease, first attacks (ie, vestibular migraine), and peripheral dizziness (ie, vestibular neuritis and stroke) in cases of acute dizziness. Currently, the Head Impulse, Nystagmus, and Test of Skew are recommended to differentiate vertigo from stroke [36]. Some studies have used telemedicine in emergency settings for medical care coordination of acute vertigo [37,38]. This study will contribute to organizing the existing practice and clarifying the possibilities and challenges of combining the two in considering a system of telemedicine using smartphones and devices in vertigo practice.

No previous study has reviewed the use of smartphones for vertigo telemedicine. We believe that the expansion of vertigo telemedicine based on this study will lead to increased opportunities to see patients, reduced treatment and travel time, reduced risk of infection, and improved quality of care. It also clarifies issues such as limitations in the acute setting and app combinations, which are crucial for achieving an appropriate comprehensive vertigo treatment system. A strength of this review was that we used an acknowledged framework for conducting scoping reviews, in addition to the PRISMA-ScR for guiding the reporting of the review. In addition, a broad, comprehensive, and systematic search was performed to identify published studies. To our knowledge, this review is the first to examine the characteristics of smartphones and smart devices to support dizziness telemedicine inclusively. Nevertheless, this review has some limitations. Searching the literature for “smartphone-assisted dizziness therapy” was difficult due to the diversity in the language used to describe such materials and interventions. Second, some articles may have been missed. Although multiple reviewers extracted data from included articles, unclear descriptions may have arisen.

### Conclusions

This review evaluated the use and benefits of digital technologies, such as smartphones and apps, in the treatment of vertigo, highlighting the potential of telemedicine in enhancing care quality and building patient trust. Despite the increased adoption of these technologies, further improvements in individual and combined systems are needed for better accuracy.

## Appendix

Multimedia Appendix 1 The aim and summary of reviewed studies.

## Abbreviations

DSS: decision support system
HIT: head impulse test
ICT: information communications technology
PRISMA-ScR: Preferred Reporting Items for Systematic Reviews and Meta-Analyses
SVV: subjective visual vertical
VR: virtual reality

## References

[1] Murdin L, Schilder AGM (2015). Epidemiology of balance symptoms and disorders in the community: a systematic review. Otol Neurotol.

[2] Nomura A, Tanigawa T, Muto T, Oga T, Fukushima Y, Kiyosue A, Miyazaki M, Hida E, Satake K (2019). Clinical efficacy of telemedicine compared to face-to-face clinic visits for smoking cessation: multicenter open-label randomized controlled noninferiority trial. J Med Internet Res.

[3] Jaensson M, Dahlberg K, Eriksson M, Nilsson U (2017). Evaluation of postoperative recovery in day surgery patients using a mobile phone application: a multicentre randomized trial. Br J Anaesth.

[4] Aida A, Svensson T, Svensson AK, Urushiyama H, Okushin K, Oguri G, Kubota N, Koike K, Nangaku M, Kadowaki T, Yamauchi T, Chung UI (2020). Using mHealth to provide mobile app users with visualization of health checkup data and educational videos on lifestyle-related diseases: methodological framework for content development. JMIR Mhealth Uhealth.

[5] Barreto RG, Yacovino DA, Cherchi M, Nader SN, Teixeira LJ, da Silva DA, Verdecchia DH (2021). The role of the smartphone in the diagnosis of vestibular hypofunction: a clinical strategy for teleconsultation during the COVID-19 pandemic and beyond. Int Arch Otorhinolaryngol.

[6] Tricco AC, Lillie E, Zarin W, O'Brien KK, Colquhoun H, Levac D, Moher D, Peters MDJ, Horsley T, Weeks L, Hempel S, Akl EA, Chang C, McGowan J, Stewart L, Hartling L, Aldcroft A, Wilson MG, Garritty C, Lewin S, Godfrey CM, Macdonald MT, Langlois EV, Soares-Weiser K, Moriarty J, Clifford T, Tunçalp Ö, Straus SE (2018). PRISMA extension for Scoping Reviews (PRISMA-ScR): checklist and explanation. Ann Intern Med.

[7] Arksey H, O'Malley L (2005). Scoping studies: towards a methodological framework. Int J Soc Res Methodol.

[8] Martin EC, Verkaik R, Stultiens JJA, van de Berg MR, Janssen AML, Leue C, Delespaul P, Peeters F, Widdershoven J, Erdkamp A, van de Weijer SCF, Blom H, Zwergal A, Grill E, Guinand N, Perez-Fornos A, Tse D, van de Berg R (2022). The DizzyQuest: relation between self-reported hearing loss, tinnitus and objective hearing thresholds in patients with Meniere's disease. J Neurol.

[9] Kıroğlu M, Dağkıran M (2020). The role of mobile phone camera recordings in the diagnosis of Meniere's disease and pathophysiological implications. J Int Adv Otol.

[10] Brodsky JR, Cusick BA, Kawai K, Kenna M, Zhou G (2015). Peripheral vestibular loss detected in pediatric patients using a smartphone-based test of the subjective visual vertical. Int J Pediatr Otorhinolaryngol.

[11] Gold D, Tourkevich R, Shemesh A, Brune A, Choi W, Peterson S, Bosely J, Maliszewski B, Fanai M, Otero-Millan J, Roberts D (2019). A novel tele-dizzy consultation program in the emergency department using portable video-oculography to improve peripheral vestibular and stroke diagnosis. Neurology.

[12] Phillips JS, Newman JL, FitzGerald JE, Cox SJ (2020). Implications of vestibular telemetry for the management of Ménière’s disease—our experience with three adults. Clin Otolaryngol.

[13] Parker TM, Farrell N, Otero-Millan J, Kheradmand A, McClenney A, Newman-Toker DE (2021). Proof of concept for an "eyePhone" app to measure video head impulses. Digit Biomark.

[14] Ippisch R, Jelusic A, Bertram J, Schniepp R, Wuehr M (2022). mVEGAS—mobile smartphone-based spatiotemporal gait analysis in healthy and ataxic gait disorders. Gait Posture.

[15] van Bonn SM, Behrendt SP, Pawar BL, Schraven SP, Mlynski R, Schuldt T (2022). Smartphone-based nystagmus diagnostics: development of an innovative app for the targeted detection of vertigo. Eur Arch Otorhinolaryngol.

[16] Wagle N, Morkos J, Liu J, Reith H, Greenstein J, Gong K, Gangan I, Pakhomov D, Hira S, Komogortsev OV, Newman-Toker DE, Winslow R, Zee DS, Otero-Millan J, Green KE (2022). aEYE: a deep learning system for video nystagmus detection. Front Neurol.

[17] Riera-Tur L, Caballero-Garcia A, Martin-Mateos AJ, Lechuga-Sancho AM (2022). Efficacy of the subjective visual vertical test performed using a mobile application to detect vestibular pathology. J Vestib Res.

[18] Shah MU, Lotterman S, Roberts D, Eisen M (2019). Smartphone telemedical emergency department consults for screening of nonacute dizziness. Laryngoscope.

[19] Wengier A, Ungar OJ, Handzel O, Cavel O, Oron Y (2021). Subjective visual vertical evaluation by a smartphone-based test-taking the phone out of the bucket. Otol Neurotol.

[20] Bamiou DE, Kikidis D, Bibas T, Koohi N, Macdonald N, Maurer C, Wuyts FL, Ihtijarevic B, Celis L, Mucci V, Maes L, Van Rompaey V, Van de Heyning P, Nazareth I, Exarchos TP, Fotiadis D, Koutsouris D, Luxon LM (2022). Diagnostic accuracy and usability of the EMBalance decision support system for vestibular disorders in primary care: proof of concept randomised controlled study results. J Neurol.

[21] Tabanfar R, Chan HHL, Lin V, Le T, Irish JC (2018). Development and face validation of a Virtual Reality Epley Maneuver System (VREMS) for home epley treatment of benign paroxysmal positional vertigo: a randomized, controlled trial. Am J Otolaryngol.

[22] van Vugt VA, van der Wouden JC, Essery R, Yardley L, Twisk JWR, van der Horst HE, Maarsingh OR (2019). Internet based vestibular rehabilitation with and without physiotherapy support for adults aged 50 and older with a chronic vestibular syndrome in general practice: three armed randomised controlled trial. BMJ.

[23] Kanyılmaz T, Topuz O, Ardıç FN, Alkan H, Öztekin SNS, Topuz B, Ardıç F (2022). Effectiveness of conventional versus virtual reality-based vestibular rehabilitation exercises in elderly patients with dizziness: a randomized controlled study with 6-month follow-up. Braz J Otorhinolaryngol.

[24] Guerrero K, Umagat A, Barton M, Martinez A, Ho KY, Mann S, Hilgenkamp T (2023). The effect of a telehealth exercise intervention on balance in adults with down syndrome. J Appl Res Intellect Disabil.

[25] Soto-Varela A, Rossi-Izquierdo M, Del-Río-Valeiras M, Faraldo-García A, Vaamonde-Sánchez-Andrade I, Lirola-Delgado A, Santos-Pérez S (2021). Vestibular rehabilitation with mobile posturography as a "low-cost" alternative to vestibular rehabilitation with computerized dynamic posturography, in old people with imbalance: a randomized clinical trial. Aging Clin Exp Res.

[26] Lubetzky AV, Kelly J, Wang Z, Gospodarek M, Fu G, Sutera J, Hujsak BD (2022). Contextual sensory integration training via head mounted display for individuals with vestibular disorders: a feasibility study. Disabil Rehabil Assist Technol.

[27] Neuhauser HK (2007). Epidemiology of vertigo. Curr Opin Neurol.

[28] Yin M, Ishikawa K, Wong WH, Shibata Y (2009). A clinical epidemiological study in 2169 patients with vertigo. Auris Nasus Larynx.

[29] Wolf SR, Christ P, Haid CT (1991). "Telemetric" electronystagmography: a new method for examination of nystagmus outside the clinic. Acta Otolaryngol Suppl.

[30] Viirre E (1996). Vestibular telemedicine and rehabilitation. Applications for virtual reality. Stud Health Technol Inform.

[31] Viirre E, Warner D, Balch D, Nelson JR (1997). Remote medical consultation for vestibular disorders: technological solutions and case report. Telemed J.

[32] Hovareshti P, Roeder S, Holt LS, Gao P, Xiao L, Zalkin C, Ou V, Tolani D, Klatt BN, Whitney SL (2021). VestAid: a tablet-based technology for objective exercise monitoring in vestibular rehabilitation. Sensors (Basel).

[33] Whitney SL, Ou V, Hovareshti P, Costa CM, Cassidy AR, Dunlap PM, Roeder S, Holt L, Tolani D, Klatt BN, Hoppes CW (2022). Utility of VestAid to detect eye-gaze accuracy in a participant exposed to directed energy. Mil Med.

[34] Parker TM, Badihian S, Hassoon A, Tehrani ASS, Farrell N, Newman-Toker DE, Otero-Millan J (2022). Eye and head movement recordings using smartphones for telemedicine applications: measurements of accuracy and precision. Front Neurol.

[35] Friedrich MU, Schneider E, Buerklein M, Taeger J, Hartig J, Volkmann J, Peach R, Zeller D (2023). Smartphone video nystagmography using convolutional neural networks: ConVNG. J Neurol.

[36] Newman-Toker DE, Kerber KA, Hsieh YH, Pula JH, Omron R, Tehrani ASS, Mantokoudis G, Hanley DF, Zee DS, Kattah JC (2013). HINTS outperforms ABCD2 to screen for stroke in acute continuous vertigo and dizziness. Acad Emerg Med.

[37] Müller-Barna P, Hubert ND, Bergner C, Schütt-Becker N, Rambold H, Haberl RL, Hubert GJ (2019). TeleVertigo: diagnosing stroke in acute dizziness: a telemedicine-supported approach. Stroke.

[38] Green KE, Pogson JM, Otero-Millan J, Gold DR, Tevzadze N, Tehrani ASS, Zee DS, Newman-Toker DE, Kheradmand A (2021). Opinion and special articles: remote evaluation of acute vertigo: strategies and technological considerations. Neurol.

