# Supplementary material for: Smartphone-Assisted Medical Care for Vestibular Dysfunction as a Telehealth Strategy for Digital Therapy Beyond COVID-19: Scoping Review
Source: JMIR Mhealth Uhealth. 2023 Sep 11;11:e48638. doi: 10.2196/48638 (PMC10496931; doi:10.2196/48638)
Supplement: Multimedia Appendix 1 [file mhealth_v11i1e48638_app1.docx]

**Appendix 1.** The Aim and Summary of reviewed studies.
